# New Onset Nystagmus in a Patient with Multiple Sclerosis

**DOI:** 10.5811/cpcem.2020.10.48448

**Published:** 2020-12-28

**Authors:** Shane Daugherty, Briana King, Melody Milliron, Jestin N. Carlson

**Affiliations:** * Lake Erie College of Osteopathic Medicine, Erie, Pennsylvania; † Saint Vincent Hospital, Department of Emergency Medicine, Allegheny Health Network, Erie, Pennsylvania

**Keywords:** Balò’s, sclerosis, nystagmus

## Abstract

**Case Presentation:**

A 50-year-old male with a history of multiple sclerosis with dizziness and nystagmus presented to the emergency department. On physical exam, nystagmus was noted. Computed tomography of the head without contrast was obtained showing a low density in the left frontal lobe. During admission, magnetic resonance imaging (MRI) findings were consistent with Balò’s concentric sclerosis.

**Discussion:**

Balò’s concentric sclerosis is a rare, inflammatory demyelinating disease, often considered to be an infrequent variant of multiple sclerosis with alternating rings of healthy myelin and demyelination leading to pathognomonic findings of concentric lamella on T2 or contrast-enhanced T1 MRI imaging.

## CASE PRESENTATION

**Image 1:**
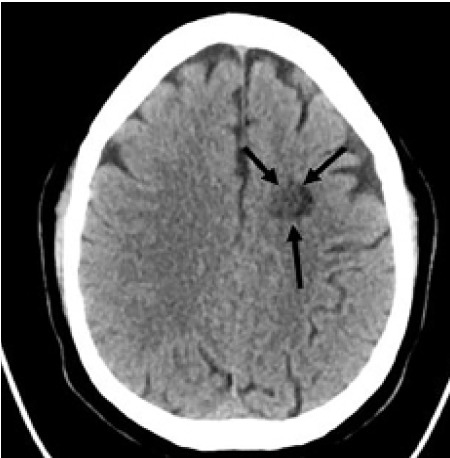
Computed tomography of the head without contrast in a 50-year-old male with dizziness. Black arrows identify an indeterminate 13-millimeter low density lesion in the left frontal lobe, further delineated by magnetic resonance imaging (Image 2).

A 50-year-old White male with a history of multiple sclerosis presented to the emergency department with fatigue, lightheadedness, and dizziness, exacerbated with sitting upright and worsening over the prior one to two days. He stated his last flare was approximately two years prior, and presented with aphasia as his primary symptom. On physical exam, the patient had non-fatigable horizontal bidirectional nystagmus with no other abnormalities noted. Non-contrast computed tomography of the head showed an indeterminate 13- millimeter low density in the left frontal lobe (Image 1).

## DISCUSSION

**Image 2:**
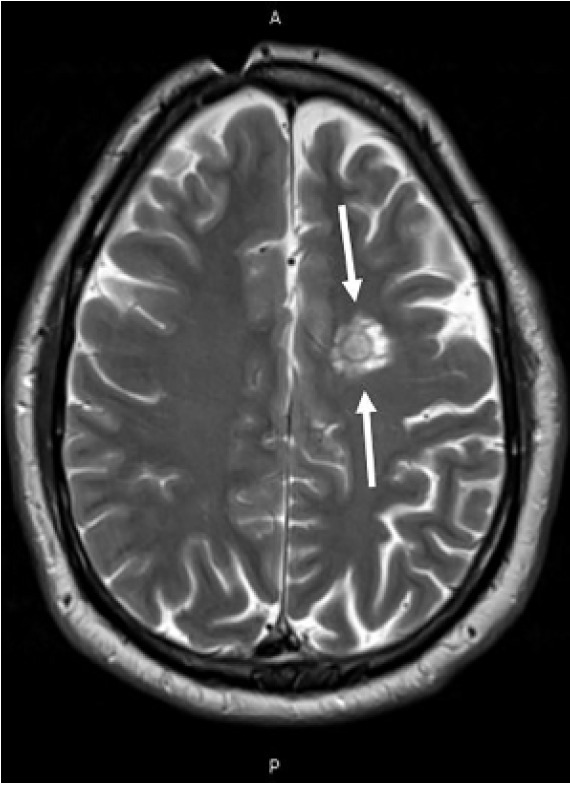
Magnetic resonance image of the head with contrast (T2 image) in a 50-year-old male with dizziness. White arrows identify a heterogeneous lesion in the left frontal centrum semiovale consistent with Balò’s concentric sclerosis.

Balò’s concentric sclerosis (BCS) is a rare inflammatory demyelinating disease, often considered to be an infrequent variant of multiple sclerosis. Initially termed leuko-encephalitis periaxialis concentrica due to its pathognomonic MRI findings, BCS was originally considered a rapidly progressive encephalopathy that was universally fatal^1^,^3^; however, it has recently been associated with spontaneous remission.^1^ Balò’s concentric sclerosis typically affects young adults with a mean age of 37 at diagnosis,^1^ and is most commonly noted in Asian and Filipino populations.^3^ Lesions are most commonly found in the cerebrum and cerebellum.^2^ Alternating rings of healthy myelin and demyelination lead to the unique and pathognomonic MRI findings of a whorled appearance of concentric lamella on T2 or contrast-enhanced T1 imaging (Image 2).^4^ The clinical presentation of BCS is heterogenous, with symptoms often indicative of a mass-like lesion depending on lesion location. Treatment options range from high-dose intravenous steroids to immunosuppressive agents.^5^ Our patient was admitted and treated conservatively with intravenous fluids, meclizine, and promethazine. He was discharged on hospital day two without complications, and was referred for outpatient vestibular rehabilitation evaluation.

**CPC-EM Capsule**

What do we already know about this clinical entity?

Dizziness is a common presenting complaint in the emergency department. Balò’s concentric sclerosis (BCS) is unlikely to be included in the differential for dizziness with nystagmus.

What is the major impact of the image(s)?

The presented images will broaden the differential diagnosis in patients with intracerebral lesions on computed tomography.

How might this improve emergency medicine practice?

Consideration of BCS in the differential could lead to a neurology rather than neurosurgical referral, allowing earlier diagnosis and treatment.
